# sesa: A Program for the Analytic Computation of Solvent‐Excluded Surface Areas**

**DOI:** 10.1002/open.202400172

**Published:** 2024-10-22

**Authors:** Lincong Wang

**Affiliations:** ^1^ The College of Computer Science and Technology Jilin University, Changchun Jilin China

**Keywords:** solvent-accessible surface, solvent-excluded surface, molecular surface, toroidal, probe-probe intersection, solvent-accessible surface area, solvent-excluded surface area

## Abstract

The surface area of a molecule, an inherent geometric property of its structure, plays important roles in its solvation and functioning. Here we present an accurate and robust program, sesa, for the analytic computation of solvent‐excluded surface (SES) areas. The accuracy and robustness are achieved through the analytic computations of all the solvent‐accessible surface (SAS) regions for a surface atom and probe‐probe intersections. The detailed comparisons of the areas for a large set of protein structures by sesa and msms, a de‐facto standard for analytic SAS and SES computations, confirm sesa’s accuracy to a good extent and in the same time reveal significant differences between them. The unprecedented accuracy and robustness of sesa make it possible to analyze in great detail the surface areas of any molecules in general and biomolecules in particular.

## Graphical abstract

sesa is an accurate and robust program for analytic computation of solvent‐excluded surface (SES) area. Its most salient feature is the computation of probe‐probe intersections by exhaustive search. Detailed comparisons with msms, the current de‐facto standard for SES area computation, show that sesa is much superior to msms.

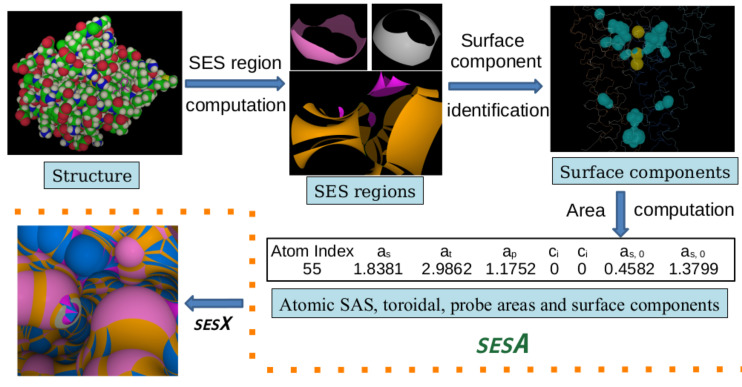

## 1 Introduction

**Figure 1:**
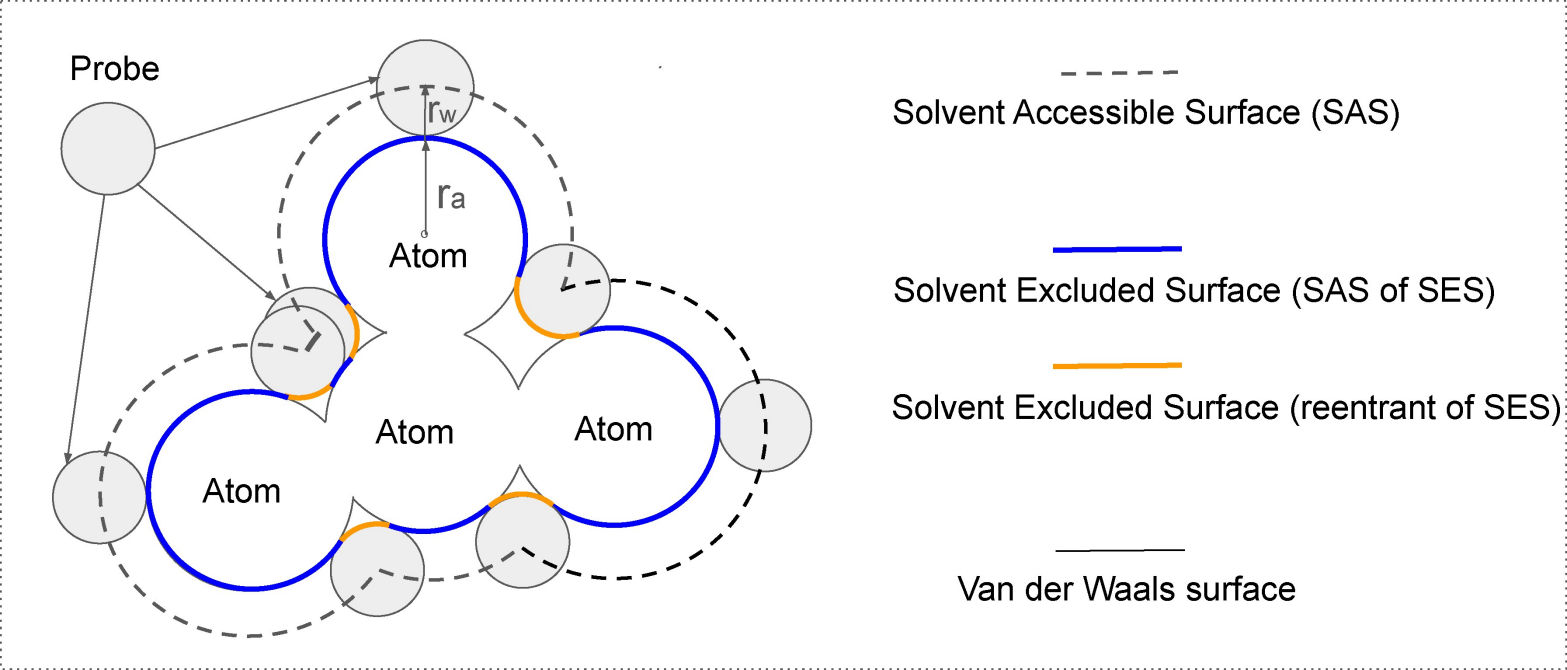
The solvent‐accessible surface (SAS), solvent‐excluded surface (SES) and van der Waals surface. A cartoon illustrates the three types of surfaces for a molecule. The SAS is traced out by the center of a rolling probe, its area is called solvent‐accessible surface area (SASA) and computed using a radius of *r_a_* +*r_w_* where *r_a_* and *r_w_* are respectively atomic and probe radii (Eq. (4)). The SES of a molecule is composed of three types of 2D patches: SAS (colored in blue), toroidal and probe (both colored in orange). A SAS patch is also called (probe) contact surface while either a toroidal patch or a probe patch is called (probe) reentrant surface. The area of the contact surface of a surface atom is a part of its atomic SES area. It is called SAS area and computed using atomic radius only (Eq. (4)). All the four atoms shown here are surface atom.

sesa is an accurate and robust program for the analytic computation of solvent‐excluded surface (SES)^1^ areas.[^1^, ^2^] The SES of a molecule consists of three types of two‐dimensional (2D) patches (Figure 1): convex spherical polygon on a solvent‐accessible (surface) atom, concave spherical polygon on a probe and toroidal patch.^[3]^ Their corresponding areas are called SAS area, probe area and toroidal area. The SES area of a molecule is the sum of its SAS, probe and toroidal areas. Though the mathematical expressions for SES area are simple and well‐known,^[3]^ the realization of analytic methods remains challenging even though algorithms for area computation have started to appear in the early seventies of last century. At present numerous programs are available for SAS or SES area estimation,^[4]^ but much less programs could compute SAS or SES area analytically.[^3^, ^5^, ^6^, ^7^, ^8^, ^9^, ^10^, ^11^, ^12^, ^13^] Furthermore, to our best knowledge, no robust programs are easily available for the analytic area computation as well as the accurate determination of both the exterior surface and the surfaces of the internal cavities of a molecule. Though msms ^[7]^ analytically computes the SES area for a molecule, can identify both the exterior surface and the surfaces of internal cavities, has been used previously as a de‐facto standard for SES area computation[^14^, ^15^, ^16^, ^17^, ^18^] and triangulation,[^20^, ^21^] and has been applied to various realms,[^22^, ^23^, ^24^, ^25^, ^26^, ^27^, ^28^] it lacks robustness especially for large‐sized molecules, and as described in the paper, is error‐prone in area computation.

In this paper, we present a novel program, sesa, that first computes all the SES regions (patches) of a molecule and then identifies, algorithmically and analytically, its exterior surface and the surfaces of its internal cavities, and finally computes, analytically, their SES areas at both (individual) atom and molecular levels. An internal cavity^2^ is inaccessible to the surrounding aqueous solvent molecules without structural changes according to the mathematical models used to represent solvent molecules and the atoms in a molecule. For brevity we call the exterior surface of a molecule *e‐surface*, the surface of an internal cavity *i‐surface*, and an internal cavity *i‐cavity*. In this paper a surface of a molecule is also called a *surface component* since it is identified as a strongly‐connected component in a graph. The correct determination of all the SES regions is a prerequisite for the correct identification of surface components, and the latter is essential for accurate area computation. In addition, particularly for a biomolecule, the two types of surfaces have different geometrical and biophysical properties, thus their separate computations may be important for a better understanding of its function. With the determination of the surface component for every SES region in a molecule, sesa proceeds to area computation. A key difference between sesa and previous algorithms is its analytic computation of probe‐probe intersections using an exhaustive search approach, which underpins its accuracy and robustness. Here a probe‐probe intersection (PPI)^3^ means the intersection of two spheres since the probes are modeled as spheres.

Except for msms, comparisons with previous (analytic) programs are tricky since they (1) use their own sets of atomic radii, (2) only accept a PDB file as input, and (3) output no (atomic) areas for individual atoms. By contrast, msms is uniquely suited for a quantitative comparison with sesa since it is able to take as inputs both atomic coordinates and radii, and it outputs atomic areas. Consequently in this paper we describe in detail the closeness and differences in SES area between sesa and msms for a set of 2,765 protein crystal structures at both atom and molecular levels. Since a toroidal patch is determined by two atoms and a probe by three, with the equal divisions of their areas among their atoms, we define the concave area of an atom as the sum of its probe area and toroidal area, and its SES area the sum of its concave area and SAS area. The comparisons between sesa and msms show that their differences in SAS area are small at molecular level^4^ and somewhat large at atom level. By contrast, their differences in concave area are large at molecular level and the largest at atom level. The analyses of their differences, particularly in concave area at atom level, suggest that the large majority of msms errors are attributable to its inaccurate treatments of PPIs. In other words, the comparisons with msms show that correct PPI computation is imperative for accurate SES area computation especially at atom level.

## Materials and Methods

### The Data Sets

We compute and compare the areas by sesa and msms for a set of 2,765 protein crystal structures downloaded from the PDB. The PDB files are preprocessed for area computation as described previously.^[29]^ Out of them there are four structures that msms fails to compute correct SAS areas for many surface atoms. The set without the four structures is denoted as ℚ (Section S6 of the Supporting Materials (SM)) with 2,761 structures and used for the comparison of the concave areas by sesa and msms. Out of ℚ there are nineteen structures for whom msms outputs a mathematically impossible SAS area for a surface atom, the set without them is denoted as ℙ with 2,742 structures, and used for the comparison of the SAS areas by the two programs. The number of atoms in ℙ is 11,285,642 with protons added using reduce^[30]^ to a PDB file that lacks of them.

### The Definitions of Atomic and Molecular SES Areas

Let **A** be the set of all the atoms in a molecule (structure).^5^ To surface atom *i* ∈ **A** we assign a nonzero SES area *a*(*i*):

(1) $$a\left(i\right)=a_{s}\left(i\right)+a_{c}\left(i\right),\;a_{c}\left(i\right)=a_{t}\left(i\right)+a_{p}\left(i\right),a_{t}\left(i\right)=\frac{1}{2}\sum_{j}a_{t}\left(i,j\right),\;a_{p}\left(i\right)=\frac{1}{3}\sum_{j,k}a_{p}\left(i,j,k\right)$$

where *a_s_* (*i*), *a_c_* (*i*), *a_t_* (*i*) and *a_p_* (*i*) are respectively the SAS, concave, toroidal and probe areas for atom *i*, *a_t_* (*i*,*j*) is the area of a toroidal patch determined by atoms *i*, *j*, and *a_p_* (*i*,*j*,*k*) the total area of spherical polygons on a probe determined by atoms *i*, *j*, *k* and all of its intersecting probes. Though it has not been explicitly stated we believe that the same expressions are used by msms to compute its atomic SES area. At atom level msms outputs only *a*(*i*) and *a_s_* (*i*). As detailed later, since *a_t_* s are computed analytically in both sesa and msms, and their differences in *a_s_* are relatively small, their differences in *a_c_* (*i*)=*a*(*i*)−*a_s_* (*i*) originate predominantly from their differences in *a_p_*.

The total SAS area, *A_s_*, and the total concave area, *A_c_*, for a surface component are defined as follows.

(2) $$A_{s}=\sum_{i=1}^{i=|C_{j}|}a_{s}\left(i\right),\;A_{c}=\sum_{i=1}^{i=|C_{j}|}a_{c}\left(i\right)$$

where the summation is over all the atoms of a surface component, *C_j_*, in a molecule. The comparisons between sesa and msms described in this paper focus on the *a_s_*, *a_c_*, *A_s_* and *A_c_* values for the e‐surface of a molecule.^6^

In sesa *a_p_* and *a_s_* are computed in two steps. The areas of their corresponding spherical polygons on a unit sphere, *u_p_* and *u_s_*, are first computed using the Gauss–Bonnet theorem with Euler‐Poincare characteristic. Given *u_p_*, *a_p_* is then computed as follows.

(3) $$a_{p}=u_{p}\times r_{w}^{2}$$

where *r_w_* is the probe radius. Given *u_s_* (*i*) for atom *i*, *a_s_* (*i*) is then computed as follows using either only *r_i_* (the radius of atom *i*) or *r_i_* +*r_w_*.4

(4) $$a_{s}\left(i\right)=u_{s}\left(i\right)\times r_{i}^{2}\;\mathrm{or}\;a_{s}\left(i\right)=u_{s}\left(i\right)\times {\left(r_{i}+r_{w}\right)}^{2}$$

The *a_s_* (*i*) computed using the second expression in Eq. (4) is generally called *atomic solvent‐accessible surface area* (*SASA*),^7^ and the summation of the *a_s_* (*i*)s for a surface component thus computed *molecular SASA* (Figure 1). For ease of exposition, in this paper, both of the *a_s_* (*i*)s in Eq. (4) are called atomic SAS areas, and their corresponding *A_s_* s molecular SAS areas.

### The SES Region Computation, Surface Component Identification and Area Computation

sesa goes through the following three steps to compute SES area: (1) the computation of SES regions, (2) the identification of surface components, and (3) area computation. A SES region could be either a spherical cap, or a spherical polygon or a toroidal patch. Each surface, be it an e‐surface or an i‐surface, consists of a set of regions.

sesa begins with the computation of all the SAS regions for every surface atom. In the following unless otherwise stated atom means surface atom. A SAS region could be either a spherical cap or a spherical polygon. An atom may have up to seven SAS regions with the exact number depending possibly on numerical accuracy. Next sesa computes the probes and toroidal patches for the entire molecule. A probe is specified by three SAS regions and a toroidal patch by two SAS regions and two probes. A probe itself may have several regions, each of them just a spherical polygon possibly with inner circles or holes. sesa then identifies the surface components of a molecule using a graph, **G**_*r*_, constructed using the set of all the SAS regions and the set of all the probes. Specifically there is a one‐one correspondence between the set of surface components of a molecule and the set of strongly‐connected components in **G**_*r*_. Finally sesa computes both atomic and molecular areas. Given a SES region, it is straightforward to compute its area analytically. For example, given the vertices and arcs of a spherical polygon, its area could be computed using the well‐known Gauss–Bonnet theorem with Euler‐Poincare characteristic. The atomic and molecular areas are just the sums of their consitituent region areas. In the following we describe each of the three steps in more detail.

#### The Computations of SAS Regions and Probes and Toroidal Patches

An atom in a molecule could have several SAS regions all accessible to solvent molecules. There exist three types of SAS regions according to their boundaries: (1) spherical cap defined by a small circle, (2) spherical polygon defined by a cycle of alternative vertices and arcs (sides), and (3) spherical band whose boundary is determined by two polygons and whose area is obtained by the subtraction of their areas. Mathematically the difference between a single polygon and a band is indicated by their different Euler‐Poincare characteristics. The key idea for the computation of the individual SAS regions of atom *i* is to find all the (shortest) cycles over the graph, **G**_*s*_(*i*), with circle‐circle intersection points as its nodes and arc lengths as its edge weights. A circle here means an intersecting circle between *i* and one of its neighboring atoms with each modeled as a sphere of radius *r*=*r_a_* +*r_w_* where *r_a_*, *r_w_* are respectively atomic and probe radii. Any circle that intersects with no other circles corresponds to a spherical cap. The set of all the SAS regions of a molecule is denoted as **R**. Since one atom could have several SAS regions, a map **M**_*a*_ with all the surface atoms as its keys and the lists of their SAS regions as values is constructed to facilitate atomic SES area computation.

Briefly the computations of probes and toroidal patches proceed as follows. A map, **M**_*r*_, with **R** as its keys and as its values the lists of the region indices in the order returned from the search over **G**_*s*_s, is first constructed and then used to compute all the probes. Specifically, each key *r_i_* in **M**_*r*_ together with two consecutive regions *r_j_* and *r_k_* in its value determine a probe represented as *p*(*r_i_*, *r_j_*, *r_k_*). The set of all the probes for a molecule is denoted as **P**. A pair of SAS regions, (*r_i_*, *r_j_*), and a pair of probes, (*p*(*i*, *j*, *k*_1_), *q*(*i*, *j*, *k*_2_)), that share the two regions, determine a toroidal patch *t*(*r_i_*, *r_j_*, *p*, *q*). The set of all the toroidal patches for a molecule is denoted as **T**.

#### The Identification of Surface Components and the Computation of SAS Areas

Except for rare cases the total surface of a molecule is composed of possible dozens of surface components. Here a surface component means that except for spherical caps, all the regions of a component could be traced out by a single imaginary rolling sphere. Surface component identification is based on the following key observation: not only could an atom have more than half a dozen SAS regions, its regions may belong to different components. For the purpose of component identification a region is represented by a class that includes data members for the atom to which it belongs, the indices of all the probes with the current region as one of their three regions, the indices of all the toroidal patches with the current region as one of their two regions, and finally a component index whose assignment is described later.

Given set **R** of all the SAS regions, set **P** of all the probes and set **T** of all the toroidal patches of a molecule as computed above, sesa then constructs a graph **G**_*r*_ with **R** as its node list. An edge exists between two nodes (regions) if they belong to the same probe. Consequently each surface component of a molecule could be readily identified as a strongly‐connected component of **G**_*r*_. **G**_*r*_ construction and component identification proceed as follows.

1. Let map **M**=θ {A map used to avoid multiple edges between any two nodes}
2. Let list **E**=θ {The edge list of **G**_*r*_}
3. Initialize a node list **N** of |**R**| elements {Region indices are saved as node data}
4. For each probe in **P**
5.  For each (*r_i_*, *r_j_*) of its three region index pairs {*r_i_* and *r_j_* are region indices}
6.   If (*r_i_*, *r_j_*) ∉ **M**
7.   Insert (*r_i_*, *r_j_*) to **M**
8. Copy the keys of **M** to **E**
9. Find the set of the strongly‐connected components, **C**, in **G**_*r*_(**N**, **E**)
10. Sort **C** according to size

The component with the largest number of nodes corresponds to the e‐surface, while all the other components are i‐surfaces. The elements of a component in **C** are node indices.^8^ Given a node index *n*(*i*), one could easily obtain its corresponding region index *r_i_* from the node data saved in **N**[*n*(*i*)]. Every SAS region, probe and toroidal patch is assigned as follows to one and only one component.

1. For each component *c*(*i*) ∈ **C** ^9^ {*c*(*i*) is component index}
2.  Assign *c*(*i*) to all the regions in **C**[*c*(*i*)] {Region index is stored as node data}
3. For each probe in **P**
4.  Set the *c*(*i*) of any of its three region indices as the probe's component index
5. For each toroidal patch in **T**
6.  Set the *c*(*i*) of any of its two region indices as the patch's component index

Finally, given **M**_*a*_ and **C** (the set of surface components) the SAS area for atom *i*, *a_s_* (*i*), is computed as follows.

1. For each region *r_i_* ∈ **C**[*c*(*i*)]
2.  Compute region area *a_s_* (*r_i_*) {Gauss–Bonnet theorem}
3. For each key *i* ∈ **M**_*a*_ {The keys in **M**_*a*_ are atom indices}
4.  Set $a_{s}\left(i\right)=\sum_{l(i)}a_{s}\left(r_{i}\right)$ {*l*(*i*) is the value for key **M**_*a*_[*i*], a list of region indices}

The summation at step 4 is over a list of the regions for atom *i* belonging to component *c*(*i*), possibly a sublist of *l*(*i*). The SAS area of a component, *A_s_*, is just the sum of the *a_s_* s computed at step 4.

#### The Computations of PPIs, and of the Areas of Probes and Toroidal Patches

A SES is composed of three types of regions: SAS region, toroidal patch and probe region. The analytic computations of both regular and spindle toroidal areas are easy and have been described previously.^[3]^ The region of a probe with no intersections with any other probes is just a spherical triangle and a simple formula exists for its area computation. However, complicated intersections with other probes, if exist, could make the analytical computation of probe area challenging. Two probes intersect if the distance between their centers is less than the probe diameter, i. e. 2.80 Å. Given set **P** it is straightforward to compute the list, *l_p_* (*j*), of the intersecting probes for any probe *j* ∈ **P**. A PPI produces an intersecting circle, a small circle on each of the two intersecting probes. Given the list *l_p_* (*j*) of intersecting circles, a graph, **G**_*p*_(*j*), with circle‐circle intersection points as its nodes and arc lengths as its edge weights is then constructed. As for SAS regions, the polygons of probe *j* are then identified either greedily as the (shortest) cycles with minimum total weights in **G**_*p*_(*j*) or exhaustively as the maximal probe regions with no intersections with any probe in *l_p_* (*j*). Given all the polygons for probe *j*, its probe area, *a_p_* (*j*), can be computed using Gauss–Bonnet theorem with Euler‐Poincare characteristic. The key steps of PPI computation and probe area computation are as follows.

1. For each probe *j* in **P**
2.  If *j* is an isolated probe
3.   
*a_p_* (*j*)=*a_tr_* {*a_tr_* is the area of the spherical triangle defined by the three contact points of *j*}
4.  Else
5.   Compute *l_p_* (*j*)
6.   Initialize graph **G**_*p*_(*j*)
7.   Find all the probe polygons *greedily* in **G**_*p*_(*j*)
8.   Compute area *a_gb_* (*j*) {Gauss–Bonnet theorem}
9.   Estimate area *a_es_* (*j*) {Shrake–Rupley algorithm}
10.   If |*a_gb_* (*j*)−*a_es_* (*j*)| <10^−2^
11.   
*a_p_* (*j*)=*a_gb_* (*j*)
12.   Else
13.   Find all the maximal probe polygons *exhaustively* in **G**_*p*_(*j*) {Depth‐first search}
14.   Compute *a_p_* (*j*) {Gauss–Bonnet theorem}

As presented in step 9, given *l_p_* (*j*), *a_p_* (*j*) could also be estimated by the Shrake–Rupley algorithm.^[31]^ The estimated areas are used only for checking the areas computed in step 8. Step 13 differs from step 7 in that not only the shortest cycles but all the cycles in **G**_*p*_(*j*) are exhaustively searched for in order to find the maximal probe regions that are free of any intersections with any probes in *l_p_* (*j*). In addition all the intersected inner regions of probe *j* are removed analytically at step 13. In theory the probe area computed at step 14 should be checked against *a_es_* (*j*). In practice, we find such a check unnecessary since as long as the PPIs are computed correctly, the final probe polygons with all of their intersected regions removed must be correct and their areas could be computed via Gauss–Bonnet theorem. With the surface component indices for every probe and every toroidal patch determined as described in Section “The Identification of Surface Components and the Computation of SAS Areas”, the concave area of a component, *A_c_*, could be computed using Eq. (2).

### sesa Implementation and msms Area Computation and the Radii of Atoms and Probe

sesa is a standalone program written in C++ and has been successfully tested on 2,765 protein crystal structures. Please see Section S1 of SM for the empirical running times of sesa and msms on this set. On average sesa (Figure S1a) is slightly faster than msms (Figure S1b) even though sesa computes, exhaustively, both probes and their intersections, and also estimates the areas of intersected probes by the Shrake–Ruply algorithm (Section “The Computations of PPIs, and of the Areas of Probes and Toroidal Patches”).

All the areas in this paper including those by msms are computed using a probe of 1.4 Å radius and the following set of atomic radii: C(1.70 Å), N(1.55 Å), O(1.52 Å), S(1.75 Å) and H(1.09 Å). Using structure 1bl8 as an example, the areas by msms are computed as follows: “msms *.x86*_*64Linux2.2.6.1‐probe1.4‐if1bl8.xyzr‐af1bl8.area*”. With these options (default settings) msms computes only the areas for the e‐surface of a structure.

## 2 Results and Discussion

We start with a short discussion of the pros and cons of analytic computation, then describe, also briefly, surface component identification and its biological implications. Finally we proceed to the main focus of this section: the closeness and differences in both SAS area and concave area between sesa and msms. We compare sesa solely with msms for the following reasons. At present msms is the de‐facto standard for analytic area computation and SES triangulation, and is the only analytic program that is readily available and easy to use while other programs either estimate SAS and SES areas or are difficult to access. Thus msms has not only been used to validate various approximate methods for area estimation and SES triangulation,[^11^, ^21^] but also applied to different realms such as pocket identification,^[23]^ surface characterization^[25]^ and protein design.^[32]^ Furthermore, though bug‐prone and lack of robustness, especially for large‐sized proteins, msms SES triangulation has been included as a plugin in several well‐known molecular visualization programs such as pymol,^[28]^ vmd ^[26]^ and ucsf chimera.^[27]^ Most importantly in terms of comparison msms lets users define their own set of atomic radii and outputs atomic SES area, thus making it possible to do quantitative and detailed comparisons with sesa.

### 2.1 The Advantages and Challenges of Analytic Area Computation

Analytic computation has obvious advantages. Even with the inherent errors in atomic coordinate for any structural model derived from experimental observables, the areas computed via analytic expressions include no additional uncertainties. In addition, at the similar level of accuracy, analytic area computation is much faster than any approximation. In terms of SES triangulation and visualization, the analytic computations of the vertices and arcs of an individual SES region makes it possible to visualize its exact boundary. However, with analytic computation limited numerical accuracy may become a concern and the correct computation of all the individual SES regions is essential. To verify the areas computed by sesa we compare the atomic SAS areas and individual probe areas by sesa with the estimations by the Shrake–Rupley algorithm using 40,962 uniformly‐distributed grid points on the surface of a unit sphere. The comparisons show that for the set of 2,765 structures, given the lists of atom‐atom intersections^10^ and the PPIs by sesa, the differences in both the individual SAS region areas and probe areas are all <0.2 Å^2^ (Table T2 and Figure S6 of SM).

### 2.2 The Identification of Surface Components

The correct identification of surface components is a prerequisite for both area computation and quantitative comparison with other area computation programs at both atom and molecular levels. Furthermore, the e‐surface and i‐surfaces (i‐cavities) of a biomolecule likely play different roles in its structure and functioning with the former playing an important role in its solubility and folding, and the latter contributing to structural stability and local flexibility and possibly being relevant to conformational changes upon ligand binding. In addition the large majority of i‐cavities are small with their atoms sharing the physical characteristics of solvent‐inaccessible atoms. Thus from a structure‐function perspective it is important to separate e‐surface from i‐surface. In other words, in terms of structure‐function relationship^[33]^ it is useful to compute individually the areas of e‐surface and i‐surface.

As described in next section, for the large majority of the e‐surfaces for the structures in ℙ , the SAS areas by sesa are very close to those by msms. It means that their corresponding sets of e‐surface atoms must be very close to each other. For example, as shown in Table T1 of SM, the e‐surface of 1bl8 determined by sesa agrees almost entirely with that by msms. Except for four e‐surface atoms, 1817, 3159, 4571 and 5310, with negligible SAS areas (last four rows of Table T1), exactly the same set of e‐surface atoms is identified by sesa and msms even though for fourteen e‐surface atoms msms have errors ranging from 0.457 Å^2^ to 19.980 Å^2^ (Table T1 and Figure S4 of SM). sesa in general identifies more surface atoms than msms does since sesa has a higher numerical precision (double precision) than msms (Figure S5). High numerical precision improves the accuracy of component identification (Figure S2). In addition, as shown in Figure S2, the i‐cavities identified by sesa agree largely with those by pymol. Taken together the results show that the i‐cavities in 1bl8 identified by sesa agree, largely and qualitatively, with those by pymol, and except for the above four atoms agree completely with those by msms. Furthermore, the visual inspections by sesx ^11^ of the i‐cavities for the structures in ℙ confirm the correctness of the surface components by sesa.

In sesa an i‐surface may not be the same as the surface of a buried internal cavity invisible from the exterior of a molecule. In fact, as depicted in Figures S2 and S3, the i‐surface composed of atoms, 3168, 3222, 3753 and 3824, and of probes, 4305, 4306, 4307 and 4360, is easily visible from the exterior. The criterion here is that none of the four probes could have left this i‐surface without the movements of some of the surrounding atoms.

#### 2.2.1 The Biological Significance of i‐Cavities

Since this paper focuses on SES area computation we use 1bl8, the crystal structure of a well‐known bacterial potassium channel,^[34]^ as the sole example to illustrate the functional significance of i‐cavities. An examination of the i‐cavities in 1bl8 shows that except for the small one at the top‐left (Figures S2 and S3), they are strategically placed by evolution for the passage of Ks from the extracellular to the intracellular: they are located close to where the channel is too narrow for Ks to pass. These cavities facilitate the conformational changes needed for Ks to pass.

### 2.3 The SAS Areas by sesa and by msms

The molecular SAS areas, *A_s_* s, by sesa and msms are in general rather close. By contrast, the differences in atomic SAS, *a_s_*, are much larger in the sense that $|dA_{s}|\ll \sum_{i}\left|da_{s}\left(i\right)\right|$ where $dA_{s}=A_{s,sesa}-A_{s,msms}=\sum_{i}da_{s}\left(i\right)$ , *da_s_* (*i*)=*A*_*s*,*sesa*_(*i*)−*A*_*s,msms*_(*i*), *A*_*s*,*sesa*_ and *A*_*s,msms*_ are respectively the total SAS areas for e‐surface by sesa and msms, *A*_*s*,*sesa*_(*i*) and *A*_*s,msms*_(*i*) are the SAS areas for atom *i* by sesa and msms, and the summation is over all the e‐surface atoms in a molecule. Unless otherwise stated, the atomic SAS areas in this Section are computed using the second expression in Eq. (4). In the following we start with the overall closeness and differences of their SAS areas, and then focus on msms errors in *a_s_*.

#### 2.3.1 The Closeness and Differences in SAS Area between sesa and msms

**Figure 2:**
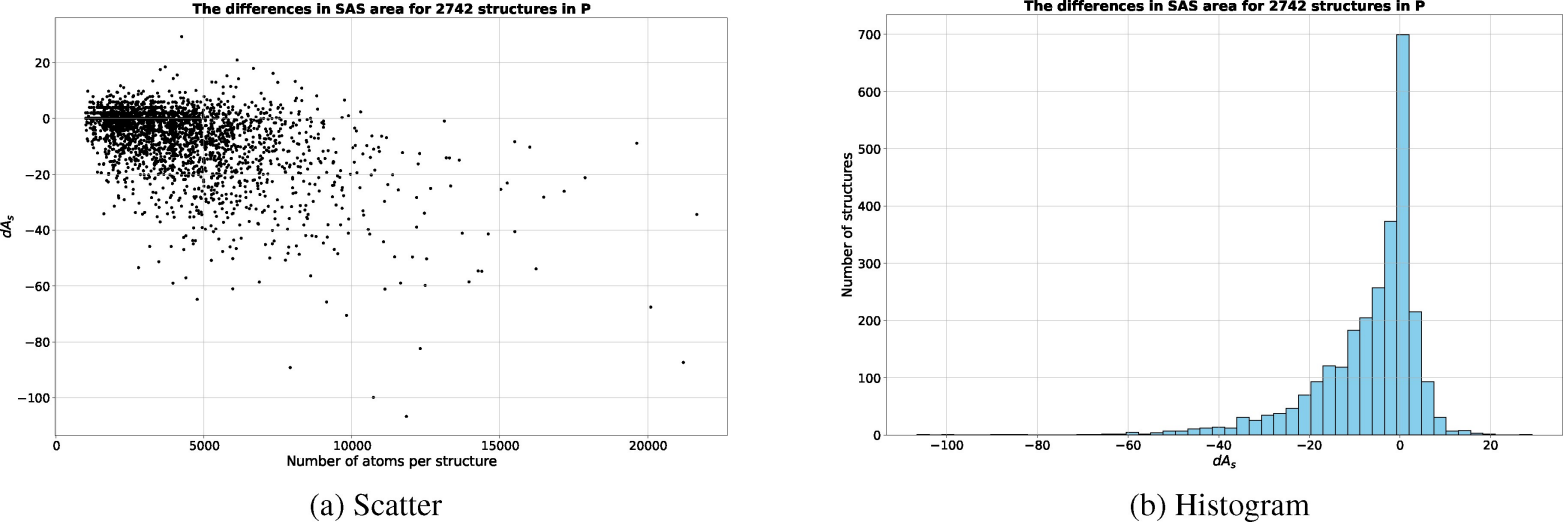
The *dA_s_* s for ℙ . (a) shows the differences in scatter plot with x‐axis being the number of atoms per structure and y‐axis *dA_s_* in Å^2^. (b) shows the differences in histogram plot with x‐axis being *dA_s_* in Å^2^.

For the large majority of the structures in ℙ , the *a_s_* s and *A_s_* s by sesa and msms agree largely with each other. As shown in Table 1 and Figure 2 at molecular level the mode for the differences, *dA_s_* s, is only −0.0024 Å^2^ while the mode for *dA_p_* s, the percentages of the difference, is 0.0359. As shown in Figure 2a there exists a weak linear correlation between the number of atoms in a molecule and *d_A_*. In terms of the number of e‐surface atoms, the differences, *d_N_* s, are also very small with a mean of 4.659 and a mode of 2.000. *d_N_* is defined as *d_N_* =*N*_*s*,*sesa*_−*N*_*s,msms*_ where *N*_*s*,*sesa*_ and *N*_*s,msms*_ are the numbers of e‐surface atoms identified respectively by sesa and msms. The smallness in both *dA_s_* and *d_N_* confirms that there exists good agreement in e‐surface between sesa and msms even though the two algorithms differ largely in e‐surface computation. msms computes e‐surface greedily using a reduced surface approach which is essentially a greedy approach, and as described later is possibly responsible for most of its errors in area computation. By contrast, sesa uses an exhaustive approach to compute the e‐surface of a molecule *after* all of its SAS regions and probes have already been computed (Section “The Identification of Surface Components and the Computation of SAS Areas”).

**Table 1:** The differences between sesa and msms in the number of surface atoms, *N_s_*, and molecular SAS area, *A_s_*, for *P*. As listed here and shown in Figure 2, on average, *N*_*s*,*sesa*_ is slightly larger than *N*_*s,msms*_ while *A*_*s*,*sesa*_ is slightly smaller than *A*_*s,msms*_.

	Minimum	Mean	Mode	Median	Maximum	Stdev
*d_N_* =*N*_*s*,*sesa*_−*N*_*s,msms*_	0	4.659	2.000	4.000	96	3.516
*dA_s_* =*A*_*s*,*sesa*_−*A*_*s,msms*_ (Å^2^)	−106.599	−6.8595	−0.0024	−2.9509	29.166	12.1287
*dN_p_* =100.0×*d_N_*/*N*_*s*,*sesa*_ (%)	0.000	0.4574	0.0000	0.3527	3.898	0.4168
*dA_p_* =100.0×|*dA_s_* |/*A*_*s*,*sesa*_ (%)	0.000	0.0640	0.0359	0.0407	0.5298	0.0690

**Figure 3:**
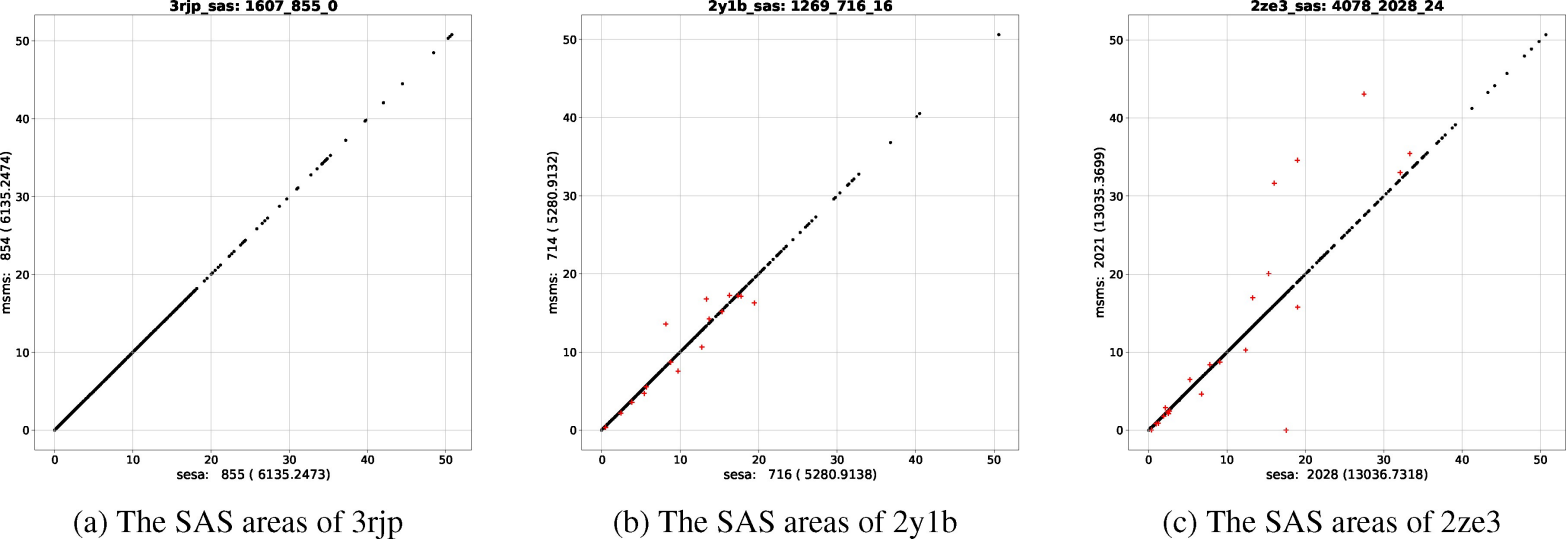
Three structures with very close *A*_*s*,*sesa*_s and *A*_*s,msms*_s. Using (a) as an example, the figure title “3rjp_sas: 1607_855_0” denotes, respectively, pdbid (3rjp), the number of atoms in a molecule (1607), *N*_*s*,*sesa*_ (855) and *N_df_* where *N_df_* is the number of the surface atoms with |*da_s_* | >0.001 Å^2^. The numbers in “sesa: 855 (6135.2473)” below the x‐axis are respectively *N*_*s*,*sesa*_ and *A*_*s*,*sesa*_, those in “msms: 854 (6135.2474)” on the left of the y‐axis are respectively *N*_*s,msms*_ and *A*_*s,msms*_. (a) shows the atomic SAS areas, *a_s_* s, by sesa and msms for 3rjp with *dA_s_* =0.00009 Å^2^ and none of the atoms having |*da_s_* | >0.00054 Å^2^. Such an excellent agreement in numerical values could only be achieved by analytic area computation using the same expressions and a very small *d_N_*. (b) shows the *a_s_* s for 2y1b: though *dA_s_* =−0.00064 Å^2^, there exists 16 atoms with |*da_s_* | >0.1 Å^2^ and 5 atoms with |*da_s_* | >1.0 Å^2^. (c) shows the *a_s_* s for 2ze3: though *dA_s_* =−1.36191 Å^2^, there exists 24 atoms with |*da_s_* | >0.1 Å^2^ and 13 atoms with |*da_s_* | >1.0 Å^2^. The x‐axis and y‐axis in each figure are, respectively, *a*_*s*,*sesa*_ and *a*_*s,msms*_. In each figure the points with |*da_s_* | >0.1 Å^2^ are depicted in red while the others in black.

In contrary to the relative closeness in *A_s_*, the differences in atomic area, *a_s_*, are in general much larger (Figure 3) in the sense that $|dA_{s}|\ll \sum_{i}\left|da_{s}\left(i\right)\right|$ . In fact, as illustrated in Figures 3b and 3c, for many structures in ℙ even though there exist a dozen or more atoms with large |*da_s_* |s, their *dA_s_* s remain small since the *da_s_* s for a molecule have, in general, both positive and negative values. Previous comparisons[^14^, ^15^, ^16^, ^18^] with msms were done only at molecular level, thus may lead to imprecise conclusions about the accuracy of their atomic areas.

#### 2.3.2. msms’s Errors in Atomic SAS Area

**Figure 4:**
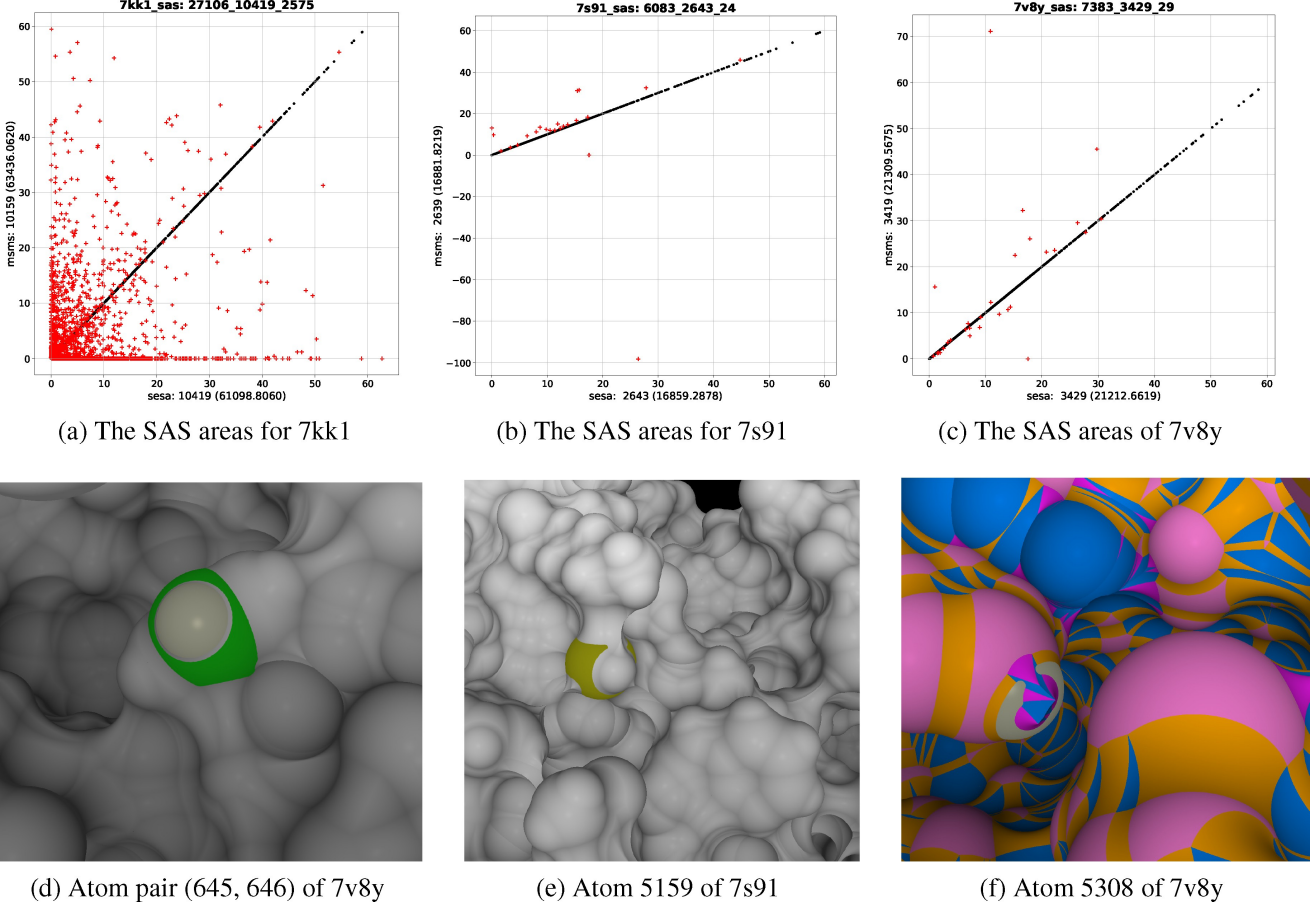
msms errors in atomic SAS area. (a) illustrates msms’s failure to compute correct SAS areas for about 1/4 of the surface atoms (2,575 out of 10,419) of 7kk1. (b) shows the SAS areas for 7 s91 by sesa and msms. (e) depicts the SAS region (colored in yellow) for atom 5159 whose *a*_*s,msms*_=−98.2577 Å^2^. The rest of the SES is colored in gray. (c) shows the SAS areas for 7v8y by sesa and msms. (f) depicts the SAS region for atom 5308 (a proton) whose *a*_*s,msms*_=71.136 Å^2^. The SAS region of atom 5308 is colored in gray with the other SAS regions, toroidal patches, spindle toroidal patches and probe polygons colored respectively in misty rose, yellow, magenta and azure. Near atom 5038 there exist sharp edges resulting from PPIs. The *a*_*s,msms*_ values for both atoms are mathematically impossible and the latter is much larger than *a*_*s*,*sesa*_ (10.873 Å^2^). The labels and numbers in (a, b, c) have the same meanings as those in Figure 3. (d) shows atom pair (645, 646) of 7v8y with 645 (F59_CE1) and 646 (F59_HE1) colored respectively in shiny gray and green, and with the rest of the SES in gray. msms excludes atom 646 but increases the SAS area for atom 645 to 32.221 Å^2^. The sesa values for atoms 645 and 646 are respectively 16.608 Å^2^ and 17.547 Å^2^ with a sum of 34.155 Å^2^, which is 1.934 Å^2^ larger than the msms value. All the SESs in the paper are generated by our SES triangulation and visualization program sesx which is capable of visualizing the exact boundaries of all the SES regions as illustrated in (d, e, f). Except for SES triangulation and visualization, the same sets of algorithms, as described here, are used by sesx for SES region and PPI computations. Since sesx triangulates each SES region individually, it could be used to verify, visually, the correctness of their computations.

msms makes four types of errors in *a_s_* computation: (1) not computing *a_s_* s for a large number of e‐surface atoms, (2) outputting mathematically impossible values, (3) outputting incorrect values assuming the correctness of sesa, (4) ignoring surface atoms with small SAS area. Type 1 errors are rare: out of the 2,765 structures msms fails to compute correct values for a large number of atoms for only four structures: 4f92, 7asw, 7kk1 (Figure 4a) and 8hgt with, respectively, 27,859, 1,759, 27,106 and 6,440 atoms with protons added. Type 4 errors are frequent but have only a minimal impact on molecular SAS area (Table T1 and Figure S5). In the following we focus on type 2 and 3 errors. With no access to msms’s source code we could not identify the exact sources of these errors. A preliminary examination shows that these errors originate likely from msms’s improper treatments of PPIs.

##### 2.3.2.1. msms Outputs Mathematically Impossible Values

Here a mathematically impossible value means either a negative value for SAS area or a value that is out of the mathematically possible range, e. g. *a_s_* >2 *π* (*r_a_* +*r_w_*)^2^ where *r_a_*, *r_w_* are, respectively, atomic radius and the probe radius.^12^ Out of 2,761 structures in ℚ , msms outputs negative *a_s_* values for two atoms: atom 5134 of 1a8d (*a_s_* =−0.0451 Å^2^) and atom 5159 of 7 s91 (*a_s_* =−98.2577 Å^2^, Figures 4b and 4e), and outputs mathematically impossible values for at least one atom in nineteen structures: 1a8s, 1dk8, 1lgp, 1o22, 2hiv, 2hxt, 2znr, 3g9x, 3gms, 3rio, 4ccw, 4maa, 7d27, 7nij, 7nyt, 7o1n, 7t40, 7v8y and 7zer (Table T3 of SM). The *a*_*s,msms*_s for these atoms are also much larger than their *a*_*s*,*sesa*_s (Figures 4c and 4f). We guess that these msms errors are likely due to its failure to compute Euler‐Poincare characteristic correctly or to treat spherical cap properly. It looks like that msms simply excludes any surface atom whose SAS region is a spherical cap (Figure 4d). The exclusion leads to an increased SAS area for its covalent‐bonded atom. Depending on the type of covalent‐bond the sum of the *a_s_* s by msms for such a pair of atoms has a fixed amount of error. For example, as shown in Figure 4d for atom pair (645, 646) of 7v8y, their *a_s_* s by msms are respectively 32.221 Å^2^ and zero with a sum 1.934 Å^2^ smaller than the sum of their *a*_*s*,*sesa*_ values.

##### 2.3.2.2. msms Outputs Erroneous Atomic SAS Areas

The most frequent msms errors are for the atoms with *a*_*s*,*sesa*_ >0, *a*_*s,msms*_ >0 and *da_s_* >0.2 Å^2^. We call them msms errors since |*da_s_* | >0.2 Å^2^ but their *a*_*s*,*sesa*_s agree, within an error of <0.2 Å^2^, with those estimated by the Shrake–Rupley algorithm^[31]^ (Table T2 and Figure S6 of SM). However it is impossible for us to pinpoint the origins of these errors with no access to msms source code. Here we use three pairs of atoms to illustrate the errors (Table 2 and Figure S7). A preliminary examination of the atoms in ℙ with such errors indicates that near these atoms there exist probe regions with either inner circles or holes or sharp edges resulting from their intersections with other probes (Figure S7). We think that the most likely culprit is the inappropriate treatments of the PPIs by msms.

**Table 2:** The large errors in atomic SAS area by msms for 1eh3, 3hr8 and 7ksf. The columns list, respectively, pdbids, atom indices, *a*_*s*,*sesa*_s, *a*_*s,msms*_s and their differences *da_s_* s where *da_s_* =*a*_*s*,*sesa*_−*a*_*s,msms*_. For the six atoms their *a*_*s,msms*_s are much larger than their *a*_*s*,*sesa*_s. Their SAS regions are depicted in Figure S7. The unit for all the areas in this paper is Å^2^.

pdbid	atomindex	*a*_*s*,*sesa*_	*a*_*s,msms*_	−*d_a_*		atomindex	*a*_*s*,*sesa*_	*a*_*s,msms*_	−*d_a_*
1eh3	1298	0.723	16.187	15.464		2866	0.322	8.308	7.986
3hr8	69	10.667	20.972	10.305		563	1.475	18.517	17.042
7ksf	1444	0.262	18.120	17.858		6914	3.545	11.740	8.195

### 2.4 The Comparisons of the Concave Areas by sesa and msms

A key feature of sesa is that the analytic PPI computation step is performed after all the SAS regions, toroidal patches and probes for all the surface components of a molecule have been computed. A probe area in sesa is defined as what remains after all of its inner regions or side arcs intersected by any other probes having been removed.^13^ By contrast, likely because of its reduced surface approach, for an e‐surface probe msms seems only to remove the regions intersected by other e‐surface probes, and furthermore, it does so in an inconsistent and erroneous way. In the following we start with the closeness in concave area between sesa and msms, and then focus on their differences especially at atom level. As detailed later the large majority of the differences in atomic concave area, *a_c_*, could be attributed to the failures of msms to properly treat PPIs. Similar msms failures have also been reported previously.[^16^, ^18^, ^19^]

#### 2.4.1 The Overall Closeness in Concave Area between sesa and msms

**Figure 5:**
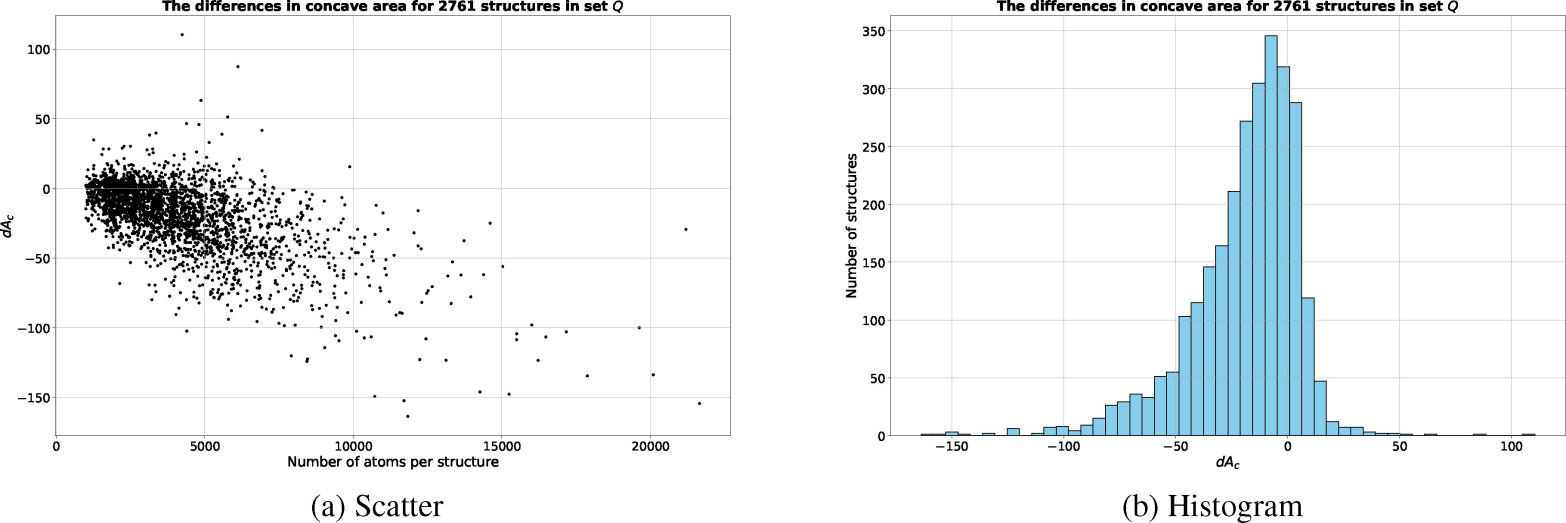
The differences in molecular concave area between sesa and msms for ℚ . (a) shows the differences in scatter plot with x‐axis being the number of atoms per structure and y‐axis *dA_c_* in Å^2^. (b) shows the differences in histogram plot with x‐axis being *dA_c_* in Å^2^.

The differences, *dA_c_* s, in molecular concave area between sesa and msms remain small particularly in terms of percentage (Table 3), and increase somewhat linearly with the number of atoms in a molecule (Figure 5a). As shown in Figure 5b, there are 235 structures in ℚ with *dA_c_* <2.0 Å^2^. Such small differences for so many structures are only possible if their *a_c_* s are computed using the same expressions as in Eqs. (1) and (2). It is an imperative for a quantitative comparison that the areas are computed using the same expressions.

**Table 3:** The differences between sesa and msms in *A_c_* for ℚ . As listed here and also shown in Figure 5, on average *A*_*c*,*sesa*_<*A*_*c,msms*_. The differences in percentage, *dC_p_*, are very small.

	Minimum	Mean	Mode	Median	Maximum	Stdev
*dA_c_* =*A*_*c*,*sesa*_−*A*_*c,msms*_ (Å^2^)	−163.7636	−19.2538	−4.9889	−14.0244	110.4421	24.4412
*dC_p_* =100.0×|*dA_c_* |/*A*_*c*,*sesa*_ (%)	0.0000	0.2407	0.0000	0.2261	1.4672	0.1405

#### 2.4.2 The Differences in Atomic Concave Area between sesa and msms

The differences in *a_c_* are much larger than those in *A_c_* in the sense that $|dA_{c}|\ll \sum_{i}\left|da_{c}\left(i\right)\right|$ where the summation is over all the e‐surface atoms in a molecule. Firstly it is an obvious bug of msms to output a negative *a_c_* value. Secondly, there exist two types of differences: *a*_*c*,*sesa*_<*a*_*c,msms*_ and *a*_*c*,*sesa*_>*a*_*c,msms*_. The former could be rationalized by msms’s negligence of the PPIs between an e‐surface probe and any of the i‐surface probes in a molecule while the latter results possibly from msms’s incorrect treatments of the PPIs among e‐surface probes.

##### 2.4.2.1. msms Outputs Negative Concave Areas

For 81 atoms in 67 structures in ℚ , msms outputs a negative *a*_*c,msms*_ value (Table T4 and Figure S8). As illustrated in Figure S8b, these atoms are always located near probe regions with inner circles or holes or sharp edges resulting from PPIs. With no source codes available to us we could only guess that the failure of msms to properly treat PPIs is most likely the culprit.

##### 2.4.2.2. msms’s Concave Areas Larger than those by sesa

**Figure 6:**
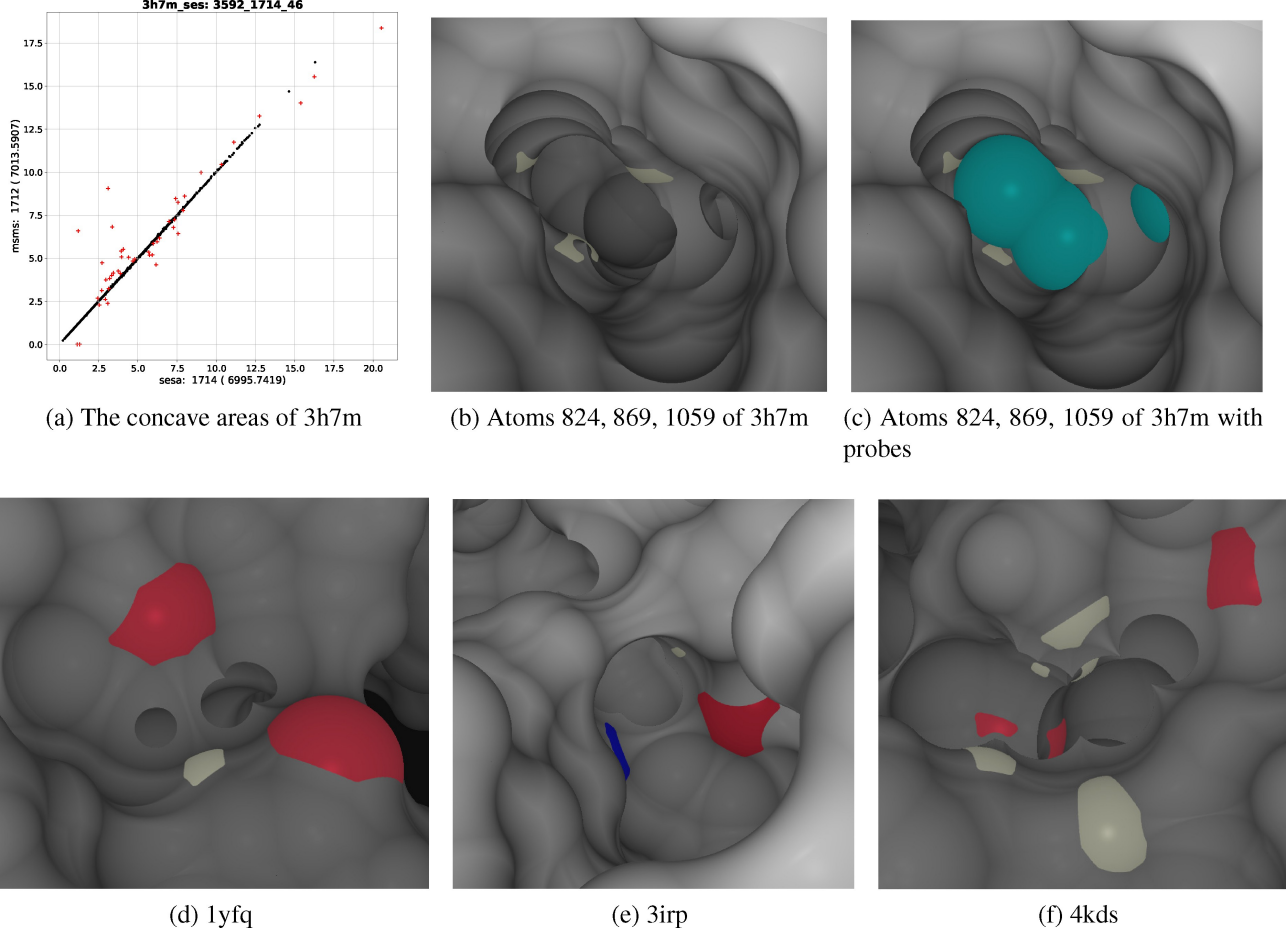
Atoms whose concave areas by sesa and msms differ largely. (a) shows the *a_c_* s for 3h7m by sesa and msms. The labels and numbers have the same meanings as those in Figure 3 except that the x‐axis and y‐axis are respectively *a*_*c*,*sesa*_ and *a*_*c,msms*_. (b, c) show the SES regions (colored in white) for atoms 824 (W104_HE1), 869 (M107_HB1) and 1059 (L119_HD13) of 3h7m. (c) also shows the probes (colored in cyan) for i‐surface 1 of 3h7m. (d) shows the SAS regions for atoms 4988 (F316_O), 5101 (Q324_OE1) and 5104 (Q324_HE22) of 1yfq. (e) shows the SAS regions for atoms 1149 (R464_NH2), 1369 (G479_O) and 1373 (S480_HA) of 3irp. (f) shows the SAS regions for atoms 3092 (K203_HD2), 3098 (K203_HZ2), 3336 (E219_OE1), 3925 (R257_HG1), 3930 (R257_HE) and 3988 (M260_O) of 4kds. Not all the SAS regions listed in Table 4 are shown here since some are covered by the others. The SAS regions for atom N, O and H are colored, respectively, in blue, red and white while the rest of the SES in each figure is colored in gray.

A probe on an e‐surface could be intersected by probes on either an e‐surface or an i‐surface. With default settings msms only computes the e‐surface of a structure. It means that msms ignores any intersection between an e‐surface probe and an i‐surface probe. As illustrated in Table 4 and Figures 6a, 6b and 6c, *a*_*c,msms*_>*a*_*c*,*sesa*_ for atoms 824, 869 and 1059 of 3h7m. Atom 824 has four SAS regions but only one of them is on the e‐surface. Both atom 869 and 1059 have one region on the e‐surface. The differences in *a_c_* could be attributed, at least partially, to msms’s negligence of the PPIs between an e‐surface probe and an i‐surface probe since their *a_s_* s for the e‐surface are the same (columns 2 and 6 of Table 4). In sesa a PPI may result in the removal of either the intersected region of the probe or a part of a side arc and consequently smaller probe area. This is consistent with the result that on average the *A*_*c*,*sesa*_s for ℚ are 19.2538 Å^2^ smaller than their *A*_*c,msms*_s (Table 3). In other words, these differences likely do not originate from bugs in msms, but rather from its default settings of not computing the i‐surfaces for a molecule.

**Table 4:** Atoms whose concave areas by sesa and msms differ largely. Columns 2, 3, 4, 5 list respectively *a_s_*, *a_t_*, *a_p_* and *a_c_* values. The numbers in bold‐face in columns 2 and 5 are their msms areas while the others are sesa areas. Columns 6, 7, 8 and 9 list all the regional *a_s_* s for an atom. The *a_s_* s in this table are computed with only atomic radii (Eq. (4)). Rows 2–4 list respectively the areas for atoms 824, 869 and 1059 of 3h7m. Atom 824 have four SAS regions belonging respectively to surface components 0, 1, 1, 1 where component 0 is the e‐surface. Atoms 869 and 1059 each has two regions belonging respectively to components 0 and 1. As listed in columns 2 and 6, for the three atoms their *a*_*s*,*sesa*_s for the e‐surface are the same as their *a*_*s,msms*_; Rows 6–8 list, respectively, the areas for atoms 4988, 5101 and 5104 of 1yfq; rows 9–11 for atoms 1149, 1369 and 1373 of 3irp; and rows 12–17 for atoms 3092, 3098, 3336, 3925, 3930 and 3988 of 4kds. As indicated by “0” in *a*_*s*,0_’s subscript, the SAS regions of the last twelve atoms are all on the e‐surfaces of their respective structures. The SES regions for the four sets of atoms are shown in Figure 6.

Atom	*a_s_*	*a_t_*	*a_p_*	*a_c_*	*a*_*s*,0_	*a*_*s*,1_	*a*_*s*,1_	*a*_*s*,1_
3h7m_824 (W104_HE1)	0.9998 (**0.3640**)	1.1678	1.9288	3.0966 (**9.0607**)	*0.3640*	0.2615	0.3741	0.0002
3h7m_869 (M107_HB1)	0.0776 (**0.0684**)	1.2043	2.1631	3.3674 (**6.8206**)	*0.0684*	0.0092		
3h7m_1059 (L119_HD13)	0.8368 (**0.0920**)	0.6037	0.5837	1.1874 (**6.5857**)	*0.0920*	0.7448		
								

Atom	*a_s_*	*a_t_*	*a_p_*	*a_c_*	*a*_*s*,0_	*a*_*s*,0_	*a*_*s*,0_	*a*_*s*,0_
1yfq_4988 (F316_O)	3.0377 (**3.0376**)	5.3604	14.0874	19.4478 (**13.1196**)	3.0242	0.0081	0.0054	
1yfq_5101 (Q324_OE1)	7.2584 (**7.2583**)	8.7523	6.5180	15.2703 (**10.7953**)	7.2584			
1yfq_5104 (Q324_HE22)	0.6397 (**0.6397**)	2.6058	8.0361	10.6419 (**6.5474**)	0.4008	0.2389		
3irp_1149 (R464_NH2)	0.3839 (**0.3839**)	3.2155	7.4986	10.7141 (**4.9154**)	0.1356	0.2483		
3irp_1369 (G479_O)	2.0368 (**2.0368**)	5.0002	8.6348	13.6350 (**10.520**4)	1.9949	0.0420		
3irp_1373 (S480_HA)	0.3491 (**0.3490**)	1.6814	6.0901	7.7715 (**1.8538**)	0.0205	0.3286		
4kds_3092 (K203_HD2)	0.2751 (**0.1726**)	1.6681	5.0518	6.7199 (**1.8133)**	0.2451	0.0299		
4kds_3098 (K203_HZ2)	0.4768 (**0.6512**)	1.8961	5.8071	7.7032 (**2.4527**)	0.4638	0.0096	0.0009	0.0025
4kds_3336 (E219_OE1)	2.2359 (**2.1274**)	4.4013	11.1848	15.5861 (**12.4055**)	2.1214	0.1145		
4kds_3925 (R257_HG1)	1.7544 (**1.4520**)	3.0941	1.7407	4.8348 (**3.5097**)	1.7544			
4kds_3930 (R257_HE)	0.7423 (**0.6681**)	2.7121	4.0436	6.7557 (**3.9120**)	0.7423			
4kds_3988 (M260_O)	1.2074 (**1.1356**)	3.5516	7.4404	10.992 (**7.6042**)	1.2037	0.0037		

##### 2.4.2.3. msms’s Concave Areas Smaller than those by sesa

All the comparisons with msms are done with its default settings: i. e. without requiring msms to compute any i‐surfaces. If both sesa and msms are correct, there should be no differences in *a_c_* for any e‐surface atom whose probes are free of PPIs, and *a*_*c,msms*_ *a*_*c*,*sesa*_ for all other e‐surface atoms. Thus it comes as a surprise to us that msms frequently outputs *a_c_* s that are smaller than those by sesa by an amount much larger than possible numerical errors, say 10^−4^ Å^2^. A preliminary examination shows that these large decreases in *a*_*c,msms*_ seem to be related to msms’s improper treatments of the PPIs between two e‐surface probes. In the following we use three sets of atoms in three structures to illustrate this type of msms errors.

As shown in rows 6, 7 and 8 of Table 4 and Figure 6d, the *a*_*s*,*sesa*_s and *a*_*s,msms*_s for the three atoms of 1yfq are almost the same. Atoms 4988 and 5104 have respectively three and two SAS regions, all of them on the e‐surface (rows 6 and 8 of Table 4) while atom 5101 have only one SAS region also on the e‐surface. Atoms 4988 and 5104 share an inner circle formed by a PPI, atoms 4988, 5101 and 5104 share an arc formed by a PPI, and atoms 4988 and 5101 also share an arc formed by another PPI. These two arcs link together to form an inner hole across the two probes. The areas of any inner circle and hole are subtracted in sesa. However, for reasons unknown to us, msms not only excludes them, but their *a_c_* s are also further reduced by a relatively large amount. As listed in rows 9, 10 and 11 of Table 4 and Figure 6e, each of the three atoms, 1149, 1369 and 1373, of 3irp has two SAS regions, both of them are on the e‐surface. There exists an inner hole formed by the PPIs among the three atoms. As listed in column 2, the *da_s_* s for the two sets of atoms are almost zero, and since *a_t_* is determined by SAS regions and all of their SAS regions are on the e‐surfaces of their respective structures, the differences in *a_c_* for the above six atoms originate predominantly from their differences in *a_p_*. The six atoms, 3092, 3098, 3336, 3925, 3930 and 3988, of 4kds (rows 12–17 of Table 4) have rather complicated SES geometry (Figure 6f). All of their SAS regions are on the e‐surface with atoms 3092, 3336 and 3988 having two SAS regions, 3098 four, and 3925 and 3930 one. Near these atoms there exist probe regions with sharp edges resulting from their intersections with other probes. In addition the *da_s_* s for some of these six atoms are relatively large.

In summary, the common feature of the atoms with *a*_*c,msms*_<*a*_*c*,*sesa*_, as illustrated above, is that near them there exist probe regions either with inner circles or holes, or sharp edges resulting from PPIs. From an algorithmic perspective, in theory, the PPIs could be discovered starting from either side but in practice with limited numerical accuracy it seems that the greedy strategy of msms may somehow result in wrong treatments for some PPIs.

##### 2.4.2.1 The Differences in *A_c_* s are Smaller than those in *a_c_* s

As illustrated in Figures 6a, S8a and S9a, the atomic *da_c_* s could be either positive or negative. Consequently the differences in *A_c_* could become small if the positive and negative values of the *da_c_* s mostly cancel each other out, i. e. $|dA_{c}|\ll \sum_{i}\left|da_{c}\left(i\right)\right|$ . This discrepancy can be illustrated with the three atoms, 909, 910, and 912 of 1lfp. As shown in Table 5 and Figure S9, the three atoms are close to each other on the e‐surface. However, for reasons unknown to us, *da_c_* =−7.6867 Å^2^ for 909 but *da_c_* =+4.4576 Å^2^ for 910 and *da_c_* =+1.6141 Å^2^ for 912. In addition, the *da_s_* s for the three atoms are quite large. Near the three atoms there exist probe regions with sharp edges resulting from their intersections with other probes. The smallness of |*dA_c_* | when compared with $\sum_{i}\left|da_{c}\left(i\right)\right|$ shows that it is important to output and compare SES areas at atom level especially for the atoms and residues in ligand‐protein binding sites^[33]^ and enzyme active sites.^[35]^ Performing comparisons with msms at molecular level only[^14^, ^15^, ^16^, ^18^] may conceal possible large differences at atom level.

**Table 5:** The differences in concave area for atoms 909, 910 and 912 of 1lfp. Atom 909 have three SAS regions all of them belong to component 0, i. e. the e‐surface of 1lfp, and atoms 910 and 912 each has a single region on the e‐surface. The table items have the same meanings as those in Table 4.

Atom	*a_s_*	*a_t_*	*a_p_*	*a_c_*	*a*_*s*,0_	*a*_*s*,0_	*a*_*s*,0_
909 (A59_CB)	0.1781 (**0.4858**)	1.0921	3.3144	4.4065 (**12.0932**)	0.0352	0.1188	0.0241
910 (A59_HB2)	1.0432 (**0.8005**)	3.3666	6.6823	10.0489 (**5.5913**)	1.0432		
912 (A59_HB1)	0.1401 (**0.0643**)	1.1634	1.8180	2.9814 (**1.3673**)	0.1401		

## 3 Conclusions

A program, sesa, has been developed for analytic SAS and SES area computations. The accuracy and robustness of sesa relies on (1) the analytic computation of all the SES regions of a molecule, (2) the accurate identification of surface components, and (3) the analytic and exhaustive computation of probe‐probe intersections. sesa has been tested on a set of 2,765 protein crystal structures and its correctness has been verified by the comparisons of the areas by sesa with both the areas estimated by the Shrake–Rupley algorithm and the areas by msms. In the same time, the detailed comparisons with msms especially at atom level reveal several types of msms errors in SAS and SES areas resulting possibly from its improper treatments of probe‐probe intersections. From an algorithmic perspective, the key differences lie in how the probes and their intersections are computed with msms taking greedy approaches while sesa taking exhaustive approaches. The unprecedented accuracy and robustness of sesa make it possible to analyze, in terms of SAS and SES, experimentally‐determined structures, predicated models and the models from molecular dynamics simulation in general and various types of ligand‐protein interfaces in particular, and by extension sesa should be useful for structure‐based drug design and protein design.

## Author Contributions

Lincong Wang: Investigation; conceptualization; writing – review and editing; visualization; validation; methodology; software.

## Conflict of Interests

The authors declare no competing interests.

4

## Data Availability

The data that support the findings of this study are openly available in the PDB.

## Footnotes

n1 Abbreviations: SES, solvent‐excluded surface; SAS, solvent‐accessible surface; SASA, solvent‐accessible surface area; esurface, the exterior surface of a molecule; i‐surface, the surface of an internal cavity; i‐cavity, internal cavity; PPI, probe‐probe interaction; 2D, two‐dimensional; PDB, Protein Data Bank; SM, Supplementary Materials.

n2 Such a cavity is sometimes called void but since it is possible for water molecules got trapped there we prefer to call it internal cavity.

n3 Probe‐probe intersection is usually called self‐intersection or singularity.[^3^, ^7^]

n4 In this paper at molecular level means mainly at surface component level. We prefer at molecular level for ease of exposition.

n5 In this paper structure and molecule are used interchangeably.

n6 For ease of exposition, we also call *A_s_* and *A_c_* molecular SAS area and molecular concave area even though they are the areas for a surface component only.

n7 In fact, msms outputs only atomic SES area and atomic SASA.

n8 A component is represented by a vector in our implementation.

n9 The same **C** is used to denote both the strongly‐connected components in **G**_*r*_ and the surface components in a molecule.

n10 An atom‐atom intersection here means the intersection of two spheres since atoms are modeled as sphere.

n11
sesx is a program written in C++/openGL/Qt for the triangulation and visualization of all the individual SES regions. It shares with sesa the set of algorithms for their analytic computations.

n12 Every structures in ℚ has been preprocessed to make sure that each atom is bonded to some others.

n13 One could argue that the removals of such inner circles or holes or sides make no much physical sense but doing so consistently and systematically is critical for sesa’s accuracy and robustness.
